# Investigating Genetic Diversity and Correlations Between Mineral Concentration and Neurotoxin (*β*-ODAP) Content in the *Lathyrus* Genus

**DOI:** 10.3390/plants13223202

**Published:** 2024-11-14

**Authors:** Fadoua Abdallah, Zakaria Kehel, Mohamed Amine El Kalchi, Ahmed Amri, Adil el Baouchi, Zine El Abidine Triqui, Moez Amri, Shiv Kumar

**Affiliations:** 1 International Center for Agricultural Research in the Dry Areas (ICARDA), Rabat 10112, Morocco; fadoua.abdallah.fadoua@gmail.com (F.A.);; 2 Faculty of Sciences, University Mohammed V, Rabat 10106, Morocco; 3 Agence National des Eaux et Forêts (ANEF), Agro Paris Tech, 75007 Paris, France; 4 AgroBioSciences Program (AgBS), College of Agriculture and Environmental Science (CAES), University Mohammed VI Polytechnic (UM6P), Ben Guerir 43150, Morocco

**Keywords:** *Lathyrus* spp., grass pea, *β*-ODAP content, neurotoxins, mineral concentration, wild crop relatives, genetic variability, correlation

## Abstract

Grass pea (*Lathyrus sativus* L.) is a nutritious legume crop well-adapted to fragile agro-ecosystems that can survive under challenging climatic conditions. The cultivation of grass pea faces stigma primarily due to the presence of *β*-*N*-Oxalyl-*L*-*α*, *β*-diaminopropionic acid (*β*-ODAP), which is associated with a risk of inducing neurolathyrism upon prolonged consumption of its grains as a staple diet. The grass pea improvement program of the International Center for Agricultural Research in the Dry Areas (ICARDA) aims to reduce *β*-ODAP content to a safe level along with improving yield potential and nutritional quality of grass pea. In this study, 183 germplasm accessions representing 13 different *Lathyrus* species and 11 *L. sativus* breeding lines were evaluated for *β*-ODAP content based on Rao protocol and mineral concentration using ICP-OES. Significant variability was observed among the accessions for the studied traits. The results showed low *β*-ODAP content and high mineral concentration in 25 accessions of crop wild relatives, which included *L. cicera*, *L. ochrus*, and *L. cassius*, with one accession IG65277 of *L. cassius*, in addition to two lines, IG117034 and ACC1335, of *L. sativus* having very low *β*-ODAP content. Furthermore, some accessions of *L. pseudocicera*, *L. aphaca*, *L. cicera*, *L. marmoratus*, *L. gorgoni*, and *L. tingitanus* also showed low *β*-ODAP content. The results showed significant positive correlations among different trait combinations, viz., K and P (r = 0.193 ***), K and Fe (r = 0.177 ***), Mn and Fe (r = 0.210 ***), Mn and Se (r = 0.137 ***), *β*-ODAP and Mg (r = 0.158 **), and *β*-ODAP and Ca (r = 0.140 **). *L. cicera*, *L. ochrus*, and *L. cassius* were identified as a great source for improving the mineral concentration and reducing *β*-ODAP content in the cultivated grass pea.

## 1. Introduction

Grass pea (*Lathyrus sativus* L.) is a nutritious legume popularly known as chickling pea, khesari in India and Bangladesh, pois carré or gesse in France, Cicerchia coltivata in Italy, guaya in Ethiopia, and san li dow in China. It is an annual cool-season crop of economic and ecological significance in South Asia and sub-Saharan Africa, and to a limited extent in Central and West Asia and North Africa (CWANA), Southern Europe, and South America [1]. Globally, grass pea cultivated area at 0.70 million ha with 0.79 million tons’ production has observed drastic reduction from the last global estimate of production (1.2 million tons) and area (1.5 million ha) [2]. Grass pea is one of the most resilient crops due to its ability to grow under extreme climatic conditions such as drought, waterlogging, soil salinity, and high temperature, but remains the most underutilized crop for adaptation to fragile agro-ecosystems [1].

The genus *Lathyrus* comprises about 181 annual and perennial species [3]. *L. sativus* is widely cultivated as a food and feed crop [4], mostly in the Indian subcontinent, Ethiopia, and the Mediterranean region [5]. *L. cicera* is grown mainly as stock feed, both as fodder and grain, and its cultivation has been confined to South-western Europe and, to a limited extent, in West Asia and North Africa [6]. Recently, grass pea has received considerable attention from scientific and farming communities, and it is considered an excellent option for building agri-food sustainability under stress conditions such as drought, waterlogging, high temperature, cold, and salinity [7]. It has a very hardy and penetrating root system and, therefore, can be grown on a wide range of soil types, including very poor soil and heavy clays. Its ability to fix atmospheric nitrogen up to 124 kg/ha, especially in dry conditions [8], makes the crop well suited for harsh conditions [9]. In South Asia, grass pea is well-suited to the rice ecosystem as it is typically broadcasted in the standing rice crop before its harvest, thriving on the residual moisture. Therefore, it makes a valuable contribution for the development of a sustainable rice production system [9,10]. Compared with the other legumes, grass pea is relatively resistant to insect pests [11,12] and diseases but highly susceptible to parasitic weeds, *Orobanche crenata* and *O. foetida* [13]. The grass pea seeds are rich in protein, micronutrients, and fiber [14,15,16]. In addition to that, it is also rich in magnesium, phosphorus, calcium, iron, zinc, manganese, and copper. Despite these advantages, its consumption has declined due to the presence of a neurotoxin known as *β*-*N*-oxalyl-*L*-*α*, *β*-diaminoproprionic acid (*β*-ODAP) in seeds and vegetative tissues that can lead to neurolathyrism if taken in large quantities continuously for long periods as a staple diet [10,17,18,19,20]. Previous studies conducted on *L. sativus* showed significant variability in seeds’ *β*-ODAP content ranging from 0.518 to 1.001 mg/100 g [21]. These levels are 2 to 5 times higher than the level presumed to be safe for human consumption [22]. Various processing methods have been suggested to reduce the *β*-ODAP content [23,24]. However, none has proven entirely effective in eliminating *β*-ODAP, including techniques such as soaking and boiling, roasting, extrusion, cooking, fermenting, and autoclaving [25]. Crop improvement efforts are currently underway, employing conventional methods to identify grass pea accessions with lower neurotoxin levels at ICARDA [26]. Exploring the inter- and intra-specific genetic diversity within the *Lathyrus* genus could serve as a valuable strategy for discovering and mining low *β*-ODAP traits. The other published materials on this topic mainly focus on other aspects of *Lathyrus sativus*. The objectives of this study were as follows: (1) to identify germplasm of *Lathyrus sativus* with low *β*-ODAP levels but high concentrations of macro- and micronutrients; (2) to evaluate the relationships among nutritional traits and *β*-ODAP content; and (3) to investigate the genetic diversity in terms of nutrient and *β*-ODAP concentrations among the wild relatives of *Lathyrus*.

## 2. Results

### 2.1. β-ODAP Content and Macro- and Micronutrient Concentrations

The MANOVA (Table 1) showed significant genotypic differences (*p* < 0.01) for macro- and micronutrients and for *β*-ODAP content among the accessions. The results showed that potassium (K) and zinc (Zn) reached the highest mean values of 2953.8 ppm and 33.4 ppm, respectively. Contrariwise, the lower concentrations have been recorded in magnesium (Mg) (1027.5 ppm) and selenium (Se) (0.1 ppm). Phosphorus (P) content varied from 953.7 (IG65224) to 3788.9 ppm (IG64807). Notably, IG64807 of *L. ochrus* exhibited a maximum value of P (Table 1). Calcium (Ca) content ranged from 688.4 ppm (IG64834) to 1678.4 ppm (IG116889). The highest Ca and K concentrations were observed in *L. sativus* (IG116889) and (IG117365), respectively; both originated from Bangladesh (Table S1). The maximum Mg concentration was observed in IG65074 (*L. cicera*). Two accessions, namely, IG117012 and ACC1916, of *L. sativus* showed the highest values of iron (Fe) (57.1 ppm) and manganese (Mn) (30.0 ppm) content. ACC1348 revealed the highest Zn (48.7 ppm) content among all the accessions. The highest concentration of Se was recorded in IG62145 (*L. odoratus*) (Table 1). Low *β*-ODAP content was found in IG65277 (0.01%), while IG65340 showed a high *β*-ODAP content of 0.3%. Indeed, this *L. cassius* accession, IG65277, originated from Syria. High heritability was recorded for Mn, Fe, Se, and *β*-ODAP contents; moderate heritability was recorded for P, K, Ca, and Mg contents; and low heritability was recorded for Zn content. Significant genotypic variation was observed for all the parameters. The coefficients of variance (CV%) and LSD (95%) showed variation in nutrient content and *β*-ODAP. The highest value of CV was recorded for Mn (122.51%). Low genotypic variance (0.02) and LSD (0.1) were observed for Se.

### 2.2. Principal Component Analysis of Macronutrients

**Figure 1:**
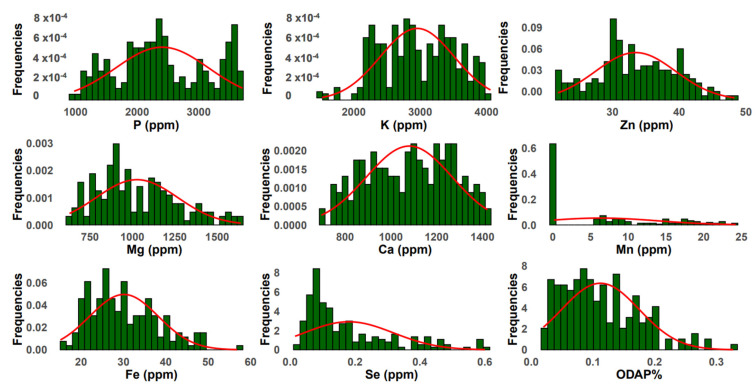
Frequency distribution of macro- and micronutrients and *β*-ODAP concentrations in grass pea species.

**Figure 2:**
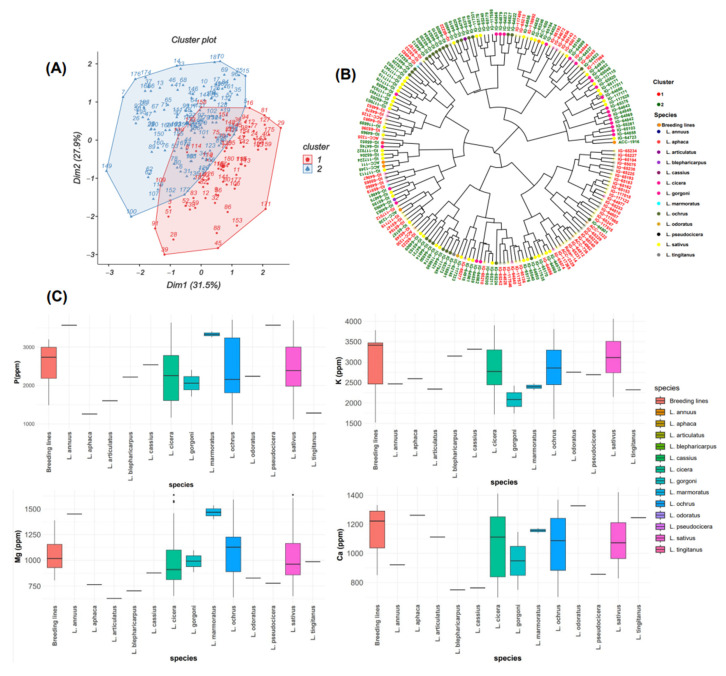
(**A**) Biplot of the first two dimensions of PCA based on the macronutrient contents for all tested grass pea germplasms. (**B**) Dendrogram showing the level of macronutrients in the total examined accessions. (**C**) Box plots showing a comparison of different macronutrient concentrations among the whole analyzed species.

Normal distribution frequency was observed for all macronutrients (Figure 1). Principal component analysis (PCA) was used to investigate the correlations among different study traits in the examined *Lathyrus* accessions. The first two principal components explained together 59.4% of the total variance for macronutrient contents (Figure 2A). The first component PC1 accounted for 31.5%, and the second component PC2 explained an additional 27.9% of the observed variation.

Table 2 revealed that PC1 was positively correlated with Mg (r = 0.599). PC2 was positively correlated with P (r = 0.630) and K (r = 0.753), but negatively correlated with Ca and Mg. PC3 recorded a positive correlation with Ca (r = 0.678), a low positive correlation with Mg (r = 0.187), and a negative correlation with P (r = −0.014) and K (r = −0.033). The results showed that PC4 was positively correlated with P (r = 0.335), Ca (r = 0.177), and Mg (r = 0.524) but negatively correlated with K (r = −0.068).

The principal component analysis biplot (Figure 2A) grouped these accessions into two clusters. The first cluster comprised 72 accessions, mainly from breeding lines *L. cicera*, *L. gorgoni*, *L. ochrus*, and *L. sativus* (Figure 2C), with high P and K and moderate Mg and Ca contents. The second cluster, consisting of 111 accessions, exhibited comparable Mg and Ca values to those in cluster 1, along with lower K and P contents than cluster 1. Cluster 2 included accessions belonging to *L. annuus*, *L. aphaca*, *L. articulatus*, *L. blepharicarpus*, *L. cassius*, *L. marmoratus*, *L. odoratus*, *L. pseudocicera*, and *L. tingitanus* (Figure 2B,C).

### 2.3. Principal Component Analysis of Micronutrients

**Figure 3:**
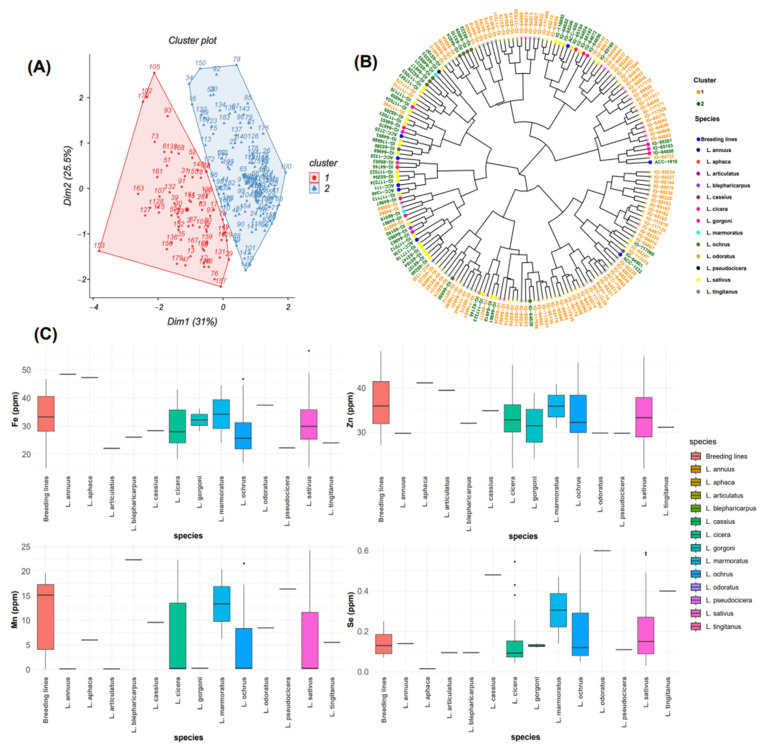
(**A**) Biplot of the first two dimensions of PCA based on the micronutrient contents for grass pea accessions. (**B**) Dendrogram showing the level of micronutrients in the total examined accessions. (**C**) Box plots showing a comparison of different micronutrient concentrations among the whole analyzed species.

The frequency distribution of the three micronutrients Zn, Fe, and Se followed a normal distribution among the tested grass pea germplasm (Figure 1). Principal component analysis for all micronutrients (Figure 3A) revealed that the first two principal components explained 56.5% of the total variation. PC1 (31%) was highly associated with Mn (r = 0.734) and Fe (0.646) and revealed positive correlation with Se (r = 0.38) and negative correlation with Zn (r = −0.19) (Table 2). A positive correlation was observed between PC2 (25.4%) and all micronutrients (Mn, Fe, and Se) except Zn (r = −0.156). A positive correlation was recorded between PC3 and Se (r = 0.717), Mn (r = 0.178), and Zn (r = 0.373), while negative correlation was revealed with Fe (r = −0.246). PC4 had negative correlations with Se (r = −0.078), Fe (r = −0.134), Mn (r = −0.141), and Zn (r = −0.416). PCA grouped all 183 accessions into two clusters comprising 114 and 69 accessions (Figure 3A). Cluster 1 grouped the accessions with high Fe and Zn concentrations. Box plots (Figure 3C) showed that cluster 1 contained breeding lines and accessions of *L. cassius*, *L. cicera*, *L. gorgoni*, *L. marmoratus*, *L. ochrus*, and *L. sativus*. The second cluster included accessions with a moderate value of Mn and Se and almost the same Zn and Fe contents (Figure 3A,B). This group embraced other species, namely, *L. pseudocicera*, *L. blepharicarpus*, *L. articulatus*, *L. annuus*, *L. tingitanus*, *L. odoratus*, and *L. aphaca* (Figure 3B,C).

### 2.4. Principal Component Analysis of β-ODAP Content

**Figure 4:**
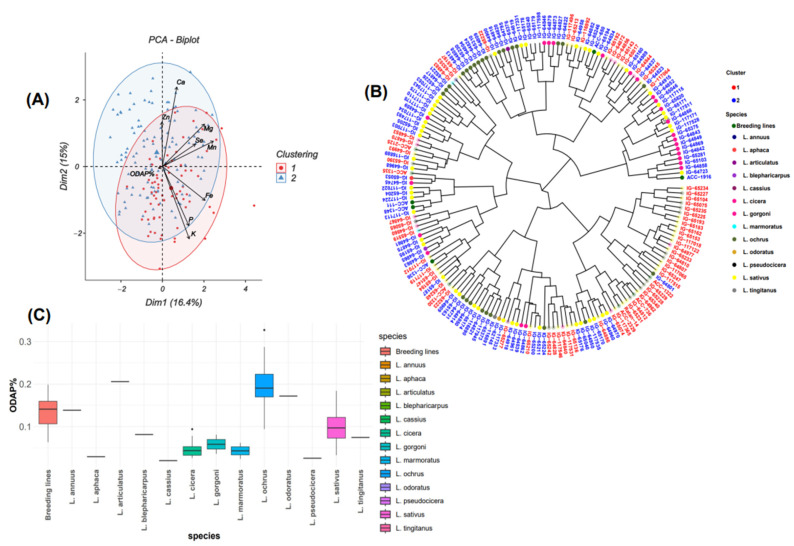
(**A**) Biplot of the first two dimensions of PCA based on *β*-ODAP content and nutrient concentration for grass pea accessions. (**B**) Dendrogram revealing the neurotoxins and nutrient levels of different tested accessions. (**C**) Box plot showing a comparison of different *β*-ODAP% concentrations among the whole analyzed accessions.

Principal component analysis indicated that the first two principal components, PC1 (16.4%) and PC2 (15%) explained together 31.4% of the total variability of *β*-ODAP content (Figure 4A). *β*-ODAP content was positively correlated with PC4 (r = 0.706) and moderately associated with PC2 (r = 0.075) and PC3 (r = 0.104). A frequency distribution representing the normal distribution of *β*-ODAP content across the tested accessions is illustrated in Figure 1. A negative correlation was observed between *β*-ODAP content and PC1 (r = −0.242) (Table 2).

Further, Biplot and ggtree (Figure 4A,B) showed 72 accessions in cluster 1 and 111 accessions in cluster 2. Clusters 1 and 2 were characterized by a high content of Zn and Fe, with moderate concentrations in Mg and Ca. On the other hand, P and K content were higher in cluster 1 than in cluster 2. On the other side, the *β*-ODAP value was 0.11% in cluster 1 and 0.10% in cluster 2. The dendrogram was used to identify and select the germplasm with good performance for nutrient and *β*-ODAP contents (Figure 4B). Three species, *L. sativus*, *L. ochrus*, and *L. cicera*, and breeding lines belonging to *Lathyrus sativus* were grouped into two clusters. Accessions belonging to *L. annuus*, *L. blepharicarpus*, *L. marmoratus*, and *L. pseudocicera* were included in cluster 1, and *L. articulatus*, *L. odoratus*, *L. cassius*, *L. aphaca*, *L. gorgoni*, and *L. tingitanus* were included in cluster 2 (Figure 4B). The box plot analysis revealed species with low and high *β*-ODAP content (Figure 4C). Accession with the lowest *β*-ODAP content belonged to *L. cassius* (0.01%, IG65277) (Table 1, Figure 4B), followed by accessions of *L. pseudocicera*, *L. aphaca*, *L. cicera*, *L. marmoratus*, *L. gorgoni*, and *L. tingitanus*. Species that revealed high *β*-ODAP concentrations (≥0.10%) were *L. ochrus* (0.3%, IG65340), *L. articulatus*, *L. odoratus*, *L. annuus*, *L. sativus*, and *L. blepharicarpus*. In general, results revealed that *L. cicera* showed the lowest *β*-ODAP content (0.02% to 0.1%), followed by *L. sativus* (0.03% to 0.2%) and *L. ochrus* (0.1% to 0.3%) (Figure 4C).

### 2.5. Correlations Among Nutrients and β-ODAP Content

**Figure 5:**
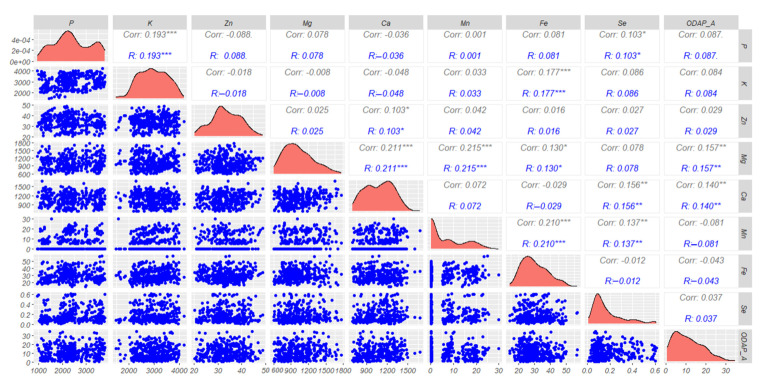
Correlogram with ggpairs among different parameters (macro- and micronutrient concentrations and percentage of *β*-ODAP) recorded for all grass pea accessions *** significant at (*p* < 0.001); ** significant at (*p* < 0.01); * significant at (*p* < 0.05).

Pairwise correlation (Figure 5) was carried out on all 183 analyzed accessions in this study. Correlation analysis indicated significantly positive associations of Mg with Mn (r = 0.215 ***) and Ca (r = 0.211 ***) (Figure 5). Significant positive correlations of K were observed with P (r = 0.193 ***) and Fe (r = 0.177 ***), and for Mn with Fe (r = 0.210 ***) and Se (r = 0.137 ***). However, low significant positive correlations were recorded for three combinations, namely, Se with P (r = 0.103 *), Zn with Ca (r = 0.103 *), and Mg with Fe (r = 0.130 *). *β*-ODAP revealed a significant positive correlation with Mg (r = 0.158 **) and Ca (r = 0.140 **). Furthermore, *β*-ODAP showed a positive correlation with P (r = 0.088), K (r = 0.085), Zn (r = 0.024), and Se (r = 0.035). The results also revealed that *β*-ODAP content was negatively correlated with Mn (r = −0.084) and Fe (r = −0.047).

### 2.6. Best-Performing Accessions

**Figure 6:**
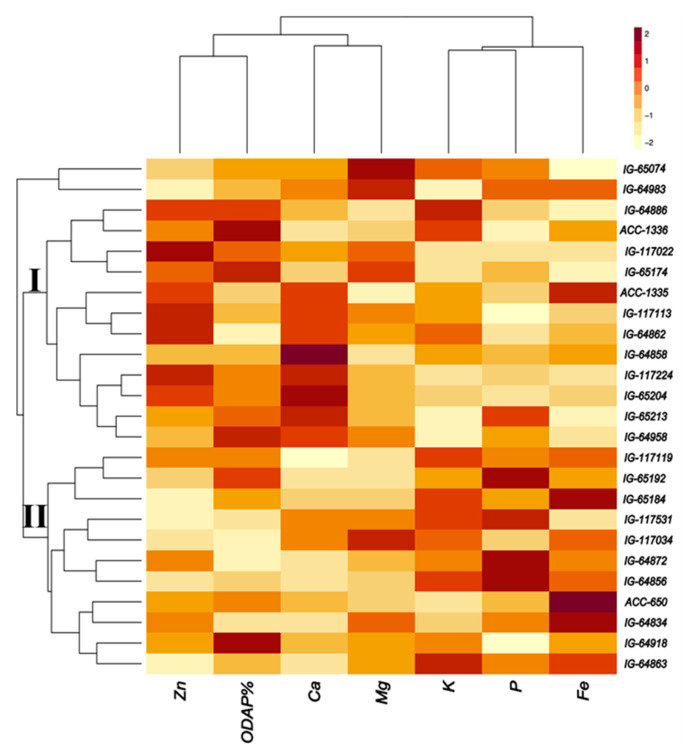
Heat map and hierarchical clustering of 25 selected grass pea accessions for the best performance parameters (high nutrient level and low *β*-ODAP content).

Twenty-five accessions were selected based on their high nutrient composition and low *β*-ODAP concentration (Figure 6). Most of these accessions represented *L. sativus*, followed by *L. cicera* and *L. marmoratus*. The first cluster contained 11 accessions with high P and Fe concentrations, such as ACC 650 (*L. sativus*), IG64834, IG64856, and IG64872 (*L. cicera*, Greece), and IG65184 and IG65192 (*L. sativus*, Ethiopia) (Figure 6). Moderate concentration was found for K in IG64863 (*L. cicera*, Greece) and for Mg in IG117034 (*L. sativus*, Bangladesh). Low *β*-ODAP content was revealed in IG64872 (*L. cicera*) from Greece and in IG117034 (*L. sativus*) from Bangladesh. In the second cluster, 14 accessions were characterized by high concentrations of Mg, Ca, and Zn. This included IG117022, IG65204, and IG65074 from *L. sativus*, IG64858 from *L. cicera*, and IG64983 from *L. marmoratus*. Furthermore, moderate content was obtained for K and Fe in *L. sativus* (IG64886) originated from Greece and in ACC1335. Two accessions revealed low *β*-ODAP in this cluster, which are IG64862 (*L. cicera* from Greece) and ACC1335.

## 3. Discussion

Traditional grass pea varieties and landraces, especially from South Asia and Sub-Saharan Africa, contain higher levels of the neurotoxin *β*-ODAP content, which can cause neurolathyrism if consumed in large quantities for prolonged periods by the undernourished population. Despite its *β*-ODAP content, grass pea is also recognized as a good source of protein, carbohydrates, and homoarginine that can sustain life during periods of famine when other food is unavailable [27]. The mineral composition of grass pea is comparable to other grain legume crops [28], although this will likely vary with soil mineral content. Global and regional efforts are underway to develop low *β*-ODAP varieties, utilizing the genetic variability available within the cultivated genepool and in the crop wild relatives. Past studies have indicated the presence of low *β*-ODAP content in the related wild species. Keeping that in mind, we analyzed 183 germplasm accessions representing 13 *Lathyrus* species originated from different continents for nutrient and *β*-ODAP contents. Results revealed significant genetic variation and high heritability for most of the traits studied. A large variation was observed for macro- and micronutrient concentrations and *β*-ODAP content.

For macronutrients, the highest values were recorded for K, followed by P, Ca, and Mg. This agrees with the findings of White et al. [28] in *L. cicera* [K (0.91%), P (0.33%), Ca (0.25%), and Mg (0.13%)] as well as in *L. sativus* [K (0.64%), P (0.42%), Ca (0.16%), and Mg (0.11%)]. In this study, the maximum values for K and Ca were observed in *L. sativus* (IG117365, IG116889, Bangladesh) and for P in *L. ochrus* (IG64807, Greece). Özyazıcı and Açıkbaş [29] reported the highest K ratio in Sel706 (2.44%), Sel1837 (2.44%), ETH-24 (2.44%), and ETH WIR-70 (2.46%) accessions. Among the studied nutrients, Zn emerged as the most abundant micronutrient, particularly in ACC1348, followed by Fe in IG117012 (*L. sativus*, Bangladesh), Mn in ACC1916, and Se in IG62145 (*L. odoratus*, Italy). Similar results were reported in *L. sativus* and *L. cicera* [28,30], in lentil [31,32], and in chickpea [33]. Lisiewska et al. [34] have reported relatively low concentrations of Mg (473–642 ppm) and Fe (20–36.7 ppm) in *L. sativus* seeds compared to what was observed in our study with Mg (1027.5 ppm) and Fe (30.1 ppm). On average, Chavan et al. [35] announced that the trace element contents for Mg and Fe of *Lathyrus maritimus* were 1800 mg/kg and 94 mg/kg, respectively, and of *L. sativus* were 1500 mg/kg and 82 mg/kg, respectively. A large number of grass pea accessions have been evaluated for nutritional value: K (8.33-11.05 ppm) [36,37], Mg (0.86–1.61 ppm), Mn (7.86–42.5 ppm) [37,38], Fe (41–73 ppm), and Zn (19–54 ppm) [16,21,36].

In the present research, lower neurotoxin concentration (*β*-ODAP) was detected in *L. cassius* with 0.01% in IG65277 (from Syria). Urga et al. [21] also reported significant variability in *β*-ODAP levels among *Lathyrus* species, with some accessions having low *β*-ODAP content considered safe for human consumption [22]. *L. cicera* cultivar “Chalus” was selected based on high yield and low *β*-ODAP (0.09%) in comparison with many other *L. cicera* accessions tested by Hanbury et al. [5]. Our results identified accessions of *L. pseudocicera*, *L. aphaca*, *L. cicera*, *L. marmoratus*, *L. gorgoni*, and *L. tingitanus* with low *β*-ODAP content. Our findings indicated that in *L. ochrus*, the *β*-ODAP content varied significantly, with IG65340 having the highest *β*-ODAP content (0.33%). Identical results were also reported by Aletor et al. [39]. In contrast to our results, Urga et al. [40] reported the highest *β*-ODAP content in *L. sativus* (IG46075) from Ethiopia. Four lines of *L. sativus*, viz., IFLLS 522 (Syria), IFFLS 588 (Cyprus), IFLLS 516 (Turkey), and IFLLS 563 (Turkey), showed low *β*-ODAP content ranging from 0.02 to 0.07%. The level presumed safe for human consumption is <0.2% [41]. As stated in our results, *β*-ODAP content ranged from 0.01% to 0.3% with an average of 0.1% for all studied species. According to Kumar et al. [2], the *β*-ODAP content of a large collection of *L. sativus* (1128 accessions) ranged from 0.150 to 0.952%, with only IG118563 (0.150%) and IG64888 (0.198%) having low content. Pandey et al. [42] have also stated that the *β*-ODAP content for 1963 entries of grass pea was between 0.067 and 0.712% and recorded that the percentage of *β*-ODAP in *Lathyrus sativus* (IPLY9, Prateek, AKL 19, BioL202, BioL203, Ratan, No. 2203, and No. 2208.) is lower than 0.10%. The range of *β*-ODAP content varied across studies. Other results have included ranges of 0.2–2% among 1000 accessions [43]; 0.02–0.74% among 81 accessions [44]; and 0.149–0.916% in a set of 150 accessions [45]. Moreover, our analysis revealed the lowest *β*-ODAP content was in *L. cicera* (0.05%), followed by *L. sativus* (0.1%) and *L. ochrus* (0.2%), which is consistent with the results of Hanbury et al. [5], Abd-El-Moneim et al. [26], and Aletor et al. [39]. Likewise, the classification was found to be identical by Eichinger et al. [46] while using capillary electrophoresis. The evaluation of 142 accessions of *L. cicera* at ICARDA during 2009 showed a range of 0.073–0.513% for *β*-ODAP content, which is much lower than the cultivated species. Therefore, *L. cicera* accessions hold promise as a source of low *β*-ODAP content in grass pea breeding programs [1]. Low *β*-ODAP was detected mainly in *L. sativus*, *L. cicera*, *L. clymenum*, *L. ochrus*, *L. hirsutus*, and *L. sylvestris*, and also with lesser content in *L. aphaca*, *L. sphaericus*, and *L. gorgoni* [47,48,49,50].

We selected the 25 best germplasm accessions with high mineral concentration and low *β*-ODAP content. The results recorded high levels of P and Fe in ACC650, IG64834, IG64856, and IG64872 (*L. cicera*) from Greece, and IG65184 and IG65192 (*L. sativus*) from Ethiopia. Furthermore, we found high levels of Mg, Ca, and Zn in certain accessions, which include *L. sativus* (IG117022, IG65204, and IG65074) from Bangladesh, Ethiopia, and Maldova, respectively; *L. cicera* (IG64858) from Greece; and *L. marmoratus* (IG64983) from Iraq. Low *β*-ODAP content was reported in *L. cicera* from Greece (IG64872, IG64862), *L. sativus* derived from Bangladesh (IG117034), and in a breeding line (ACC1335). We notice that the selected accessions differed in their origin, covering different continents (Africa, Asia, Europe, Australia). As proved by Bandana et al. [51], Prateek, Ratan, and Mahateora showed a significantly low amount of *β*-ODAP content; instead, Local Khesari and Boga Khesari revealed a very high nutritional characteristic. According to our results, Europe is the continent that contains most of the best-performing accessions, followed by Asia, Ethiopia, and then Australia. Abd-El-Moneim et al. [26] also reported that grass pea germplasm from Ethiopia and the Indian subcontinent is generally higher in *β*-ODAP (0.7–2.4%) compared to germplasm from the Near East (0.02–1.2%). This is interpreted to mean that the species’ origin influences the *β*-ODAP content in *Lathyrus*. The *β*-ODAP content in grass pea may be influenced by environment and agronomic practices [43] within the same species. Hanbury et al. [5] found for 407 *L. sativus* and 96 *L. cicera* lines collected from three geographic origins (Ethiopian, Mediterranean, European), that genotype was the most important determinant of *β*-ODAP concentration and that environment had less influence. However, Wuletaw [52] concluded for *L. sativus* that genotype and its interaction with the environment are the most significant determinants of *β*-ODAP levels.

Our research revealed that Mn was significantly and positively correlated with Fe (r = 0.210 ***) and Se (r = 0.137 ***). Zn showed moderate associations with all other micronutrients. Arslan [53] revealed a significant correlation (at 5%) between Se and Mg and a highly significant relation (at 1%) among all the other minerals. Significant and positive correlation were found in this study between Mg and Ca (r = 0.211***), while Se showed moderate relationships with P (r = 0.103*), K (r = 0.086) and Zn (r = 0.027). This study suggest that *β*-ODAP was negatively correlated with Mn (r = −0.081) and Fe (r = −0.043). Nonetheless, Basaran et al. [54] also reported that *β*-ODAP was highly correlated with Zn (r = 0.732 **) and Bore (B) (r = −0.507 *) and poorly correlated with P, K, Mg, and Fe. These latter authors indicated that interactions between *β*-ODAP and minerals are complex and vary depending on species. So, any observations regarding *β*-ODAP x mineral interaction in certain species cannot be generalized for the whole genus.

Taking into account our results, we identified grass pea germplasm with a high content of minerals and low *β*-ODAP content, which could be useful for integrating them into a genetic improvement program. Breeding for low *β*-ODAP was achieved through somaclonal mutation, which has allowed the release of several viable diploid mutants with marked alterations in plant characters [55]. The accessions of grass pea with valuable traits can also be used by the breeding programs. However, the mobilization of the genes for low *β*-ODAP and for high macro- and microelements from other *Lathyrus* species will require a lot of pre-breeding efforts. Interspecific crosses of grass pea with more than 11 *Lathyrus* species were attempted at ICARDA and allowed to develop germplasm from crosses with *L. cicera*, *L. ochrus*, *L. inconspectus*, *L. marmoratus*, and *L. hierosolymitanus* (Amri A., unpublished data). Further crosses using the identified accessions of *Lathyrus* species with low *β*-ODAP and high Zn, Fe, and Se will allow the development of high-yielding varieties with low *β*-ODAP content and biofortified for microelements.

## 4. Materials and Methods

### 4.1. Plant Material

A set of 183 germplasm accessions belonging to 13 wild *Lathyrus* species originated from different continents (Asia, Africa, Europe) and 11 *L. sativus* breeding lines were provided by the ICARDA gene bank (Table S1). During the crop seasons of 2016 and 2017, these accessions were grown under net cages to avoid outcrossing and ensure seed purification. Two samples from each accession were subjected to *β*-ODAP% and nutritional quality analysis at the ICARDA, Morocco, quality laboratory.

### 4.2. β-ODAP Protocol

Seeds harvested from a representative single plant of each accession were collected and grinded separately. A total of 5 g of powdered sample of each accession was diluted in 60% ethanol and mixed in a volumetric flask for 45 min at 200 rpm. All the tubes were centrifuged at 4500 rpm for 15 min, followed by the collection of 2 mL of supernatant in centrifuge tubes. Afterwards, 4 mL of 3 N potassium hydroxide (KOH) was added to each tube, vortexed, and placed in a boiling water bath at 100 °C for 30 min. The sample tubes were once again centrifuged at 4500 rpm for 15 min, and 250 µL of the hydrolyzed supernatant was pipetted. These samples were then combined with 750 µL of H_2_O and 2000 µL of O-phthalaldehyde (OPT) solution in tetraborate. The procedure included three blanks: 0.25 mL non-hydrolyzed extract + 0.75 mL distilled water + 2 mL of the OPA reagent (OPA blank); 0.25 mL non-hydrolyzed extract + 0.75 mL distilled water + 2 mL OPT (sample blank); and 0.25 mL hydrolyzed extract + 0.75 mL distilled water + 2 mL OPT (buffer blank). Afterward, the tubes were vortexed and incubated for 2 h at 40 °C. The spectrophotometric measurement was recorded at 425 nm with the absorbance at blank set as zero [56]. The final absorbance was given by

A = (A (sample) − A (buffer blank) − 1/3 (A (OPA blank) − A (sample blank)), (1)

A is a calibration curve made using DL-2,3-diaminopropionic acid mono hydrochloride as standard and a conversion factor to *β*-ODAP of 1.69 [39]. The general equation for calculating the concentration of *β*-ODAP is as follows:

C = ((A_3_–A_4_) − 1/3 (A_1_–A_2_) − y intercept)/Slope, (2)

where C is the concentration of *β*-ODAP in the amino acid extract; the y intercept and the slope were extrapolated from a regression line calculated from absorbance readings of D*L*-*α*-*β*-diamino propionic acid (DAP) as standard [56]. The DAP solutions were prepared and reacted with OPT precisely in the same way as described for the amino acid extracts. A1 to A4 are absorbance values for amino acid solutions.

### 4.3. Mineral Analysis

The seeds were grounded using a Cyclone mill (Twister, 10 mm–250 μm, Retsch, Éragny, France), and mineral concentration was measured using a modified HNO_3_ and H_2_O_2_ method [57]. For each accession, 500 mg of sample was placed in digestion tubes with 6 mL nitric acid (HNO_3_) at 90 °C for 60 min in the digestion block (QBlock series, Horiba, Burlington, Canada). Then, 3 mL of 30% hydrogen peroxide (H_2_O_2_) was added to each tube, followed by the second digestion when the samples were heated again at 90 °C for 15 min. Next, 3 mL of 6 M hydrochloric acid (HCl) was added to each digestion tube. Finally, all samples were cooled, filtered (Whatman No. 1 filter papers), and then diluted with distilled water to 10 mL. The mineral concentrations were carried out by inductively coupled plasma–optical emission spectroscopy (ICP-OES) (iCAP-7000 Duo, Thermo Fisher Scientific, Waltham, MA, USA). The results were based on lab references and NIST standard references using a specific calibration for the micronutrients with a dilution from 0.1 to 10 mg/L.

### 4.4. Statistical Analysis

The data were analyzed using two software tools, SPSS version 25 (IBM Corporation, New York, NY, USA) and R Core Team (2023). R: A Language and Environment for Statistical Computing. R Foundation for Statistical Computing, Vienna, Austria. https://www.R-project.org/ (accessed on 1 October 2023). Multivariate Analysis of Variance (MANOVA) for ODAP and nutrient contents was performed. Mean, max, min, R^2^, heritability, genotypic variance, coefficient of variation CV (%), and least significant difference (LSD) were determined using SPSS. All statistical descriptive parameters were tested for significance at *p* < 0.05. Principal component analysis (PCA) was performed to explore the relationships among accessions and to determine the number of clusters when accessions are associated with major nutrient concentration and low ODAP contents using ggplot, factoextra, and factoMineR in Rstudio. Hierarchical clustering analysis (HCA) was employed to form different groups of accessions based on their performance in nutrient and *β*-ODAP values, using Ward’s method based on Euclidean distance. Tree diagrams resulting from hierarchical clustering analysis were represented using the ggplot2 and ggtree packages in Rstudio. A correlogram was used to study the relationships among all studied traits with the ggpairs function. Boxplots and the frequency histograms were established with the ggplot2 package in Rstudio to provide a quick summary of the values’ variabilities.

## 5. Conclusions

This study indicates the potential of crop wild relatives as a source of novel variation for mainstreaming in the grass pea improvement program to develop improved varieties with high mineral concentration and low *β*-ODAP content. Some of the species, such as *L. cicera* (IG64872, IG64862), *L. sativus* (ACC 650, IG65184, IG65192, IG117022, IG65204, IG65074, IG117034), and *L. cassius* (IG65277), present an excellent source of desirable traits useful for enhancing the mineral concentration and reducing *β*-ODAP content in the cultivated species. *L. cassius* (IG65277 from Syria) showed the lowest *β*-ODAP content. Some of the species, namely *L. pseudocicera*, *L. aphaca*, *L. marmoratus*, *L. gorgoni*, and *L. tingitanus*, showed crossing barriers with cultivated species and require special breeding tools and techniques to overcome the bottleneck to integrating them in the grass pea improvement program.

## Figures and Tables

**Table 1:** Range, mean, F, R^2^, heritability (h^2^), coefficient of variance (CV%), genotypic variance, and LSD of nutrients and *β*-ODAP for 183 grass pea accessions belonging to 13 species.

Traits	Min.	Max.	Mean	F	R^2^	Heritability (h^2^)	CV (%)	Genotypic Variance	LSD (95%)
P	953.7 (IG65224)	3788.9 (IG64807)	2405.8	63.9 **	0.9	0.5	31.6	308,479.4	177.9
K	1478.9 (ACC1916)	4189.1 (IG117365)	2953.8	40.9 **	0.9	0.4	19.0	157,105.1	169.2
Ca	688.4 (IG64834)	1678.4 (IG116889)	1080.1	5.7 **	0.8	0.5	18.9	23,025.8	74.6
Mg	601.1 (IG64858)	1765.4 (IG65074)	1027.5	10.4 **	0.9	0.5	24.3	33,138.6	69.6
Mn	0.1 (IG117018)	30.0 (ACC1916)	6.3	21.5 **	0.9	0.6	122.5	37.5	9.6
Fe	14.4 (ACC1330)	57.1 (IG117012)	30.1	75.3 **	0.9	0.6	27.0	43.6	6.0
Zn	20.3 (IG65147)	48.7 (ACC1348)	33.4	48.4 **	0.9	0.2	18.0	8.3	8.39
Se	0.01 (IG65053)	0.6 (IG62145)	0.1	66.3 **	0.9	0.7	77.2	0.02	0.1
*β*-ODAP%	0.01 (IG65277)	0.3 (IG65340)	0.1	2.5 **	0.7	0.5	66.0	38.6	5.0

** singnificant at (*p* < 0.01).

**Table 2:** The first four principal components of principal component analyses (PCAs) for 183 grass pea accessions based on their neurotoxins (*β*-ODAP%) and macro- and micronutrient concentrations.

	PC1	PC2	PC3	PC4
P	0.044	**0.630**	−0.014	0.335
K	0.052	**0.753**	−0.033	−0.068
Ca	0.055	−0.250	**0.678**	0.177
Mg	**0.599**	−0.191	0.187	0.524
Mn	**0.734**	0.003	0.178	−0.141
Fe	**0.646**	0.252	−0.246	−0.134
Zn	−0.019	−0.156	0.373	−**0.416**
Se	0.038	0.391	**0.717**	−0.078
*β*-ODAP%	−0.242	0.075	0.104	**0.706**

Bold values for each column correspond to the parameters highly correlated (positive or negative) with each of the four PCs.

## Data Availability

The data presented in this study are available on request from the corresponding author.

## Supplementary Materials

The following supporting information can be downloaded at: https://www.mdpi.com/article/10.3390/plants13223202/s1, Table S1: *Lathyrus* accessions, their origin, and IG number included in the study for *β*-ODAP and nutrient content.
