# Supplementary material for: Investigating Genetic Diversity and Correlations Between Mineral Concentration and Neurotoxin (β-ODAP) Content in the Lathyrus Genus
Source: Plants (Basel). 2024 Nov 14;13(22):3202. doi: 10.3390/plants13223202 (PMC11598055; doi:10.3390/plants13223202)
Supplement: Supplementary file 1 [file plants-13-03202-s001.zip › plants-3279460-supplementary.pdf]

Supplementary material:

Table S1. *Lathyrus* accessions, their origin and IG number included in the study for ODAP and nutrient analysis.

Species	Geographic distribution	IG number
L. annuus (1)	Syria	65353
L. aphaca (1)		65053
L. articulatus (1)	Czech Republic	64795
L. blepharicarpus (1)		64865
L. cassius (1)	Syria	65277
L. cicera (33)	India **(1)**	64745
	Turkey **(1)**	65820
	Germany **(1)**	64879
	Syria **(1)**	65103
	Australia **(2)**	65046, 65074
	Greece **(27)**	64834, 64837, 64839, 64840, 64842, 64843, 64844, 64846, 64849, 64852, 64853, 64856, 64858, 64859, 64860, 64861, 64862, 64863, 64865, 64867, 64868, 64869, 64870, 64872, 64873, 64875, 64977
L. gorgoni (2)	Syria **(2)**	65375, 64989
L. marmoratus (2)	Iraq **(1)**	64983
	Syria **(1)**	65519
L. ochrus (42)	India **(1)**	64731
	Czech Republic **(1)**	64827
	Portugal **(1)**	64828
	Russia **(1)**	65075
	Cyprus **(11)**	64950, 65222, 65224, 65225, 65226, 65227, 65228, 65229, 65230, 65235,65242
	Syria **(7)**	65221, 65310, 65340, 65373, 65376, 65390, 117945
	Greece **(20)**	64801, 64804, 64807, 64808, 64809, 64810, 64812, 64813, 64814, 64817, 64818, 64819, 64820, 64821, 64822, 64823, 64824, 64827, 64828, 64850
L. odoratus (1)	Italy **(1)**	62145
L. pseudocicera (1)		65065
L. sativus (85)	Canada **(1)**	64993
	Bulgaria **(1)**	64900
	Iran **(1)**	65040
	Moldova **(1)**	65074
	Germany **(2)**	64915, 64918
	Greece **(2)**	64886, 64909
	Turkey **(2)**	64882, 65017
	Afghanistan **(8)**	64723, 64958, 64960, 64961, 64962, 64968, 64970, 64875
	Cyprus **(9)**	65223, 65231, 65233, 65234, 65240, 65245, 65246, 65247, 65248
	Ethiopia **(28)**	65104, 65136, 65143, 65147, 65153, 65160, 65162, 65163, 65170, 65171, 65174, 65176, 65178, 65179, 65183, 65184, 65187, 65192, 65193, 65194, 65195, 65197, 65200, 65204, 65205, 65210, 65211, 65213

	Bangladesh **(33)**	116889, 116890, 116992, 117003, 117012, 117018, 117022, 117034, 117053, 117064, 117065, 117110, 117113, 117115, 117119, 117122, 117171, 117175, 117178, 117224, 117331, 117333, 117365, 117493, 117496, 117511, 117515, 117528, 117531, 117535, 117543,117546, 117598
	India **(1)**	Ratan
L. tingitanus (1)	Bangladesh **(1)**	116891
Breeding lines (11)	ICARDA **(11)**	ACC111, ACC1322, ACC1330, ACC1335, ACC1336, ACC1348, ACC1916, ACC2125, ACC2329, ACC650, ACC736

***(..)**; Number of accessions of each species.
